# A mixed method comparison of stigma toward autism and schizophrenia and effects of person-first versus identity-first language

**DOI:** 10.3389/fpsyt.2023.1263525

**Published:** 2023-10-27

**Authors:** Desiree R. Jones, Noah J. Sasson

**Affiliations:** Department of Psychology, School of Behavioral and Brain Sciences, The University of Texas at Dallas, Richardson, TX, United States

**Keywords:** autism, schizophrenia, stigma, qualitative, terminology

## Abstract

**Introduction:**

While stigma toward autistic individuals has been well documented, less is known about how autism is perceived relative to other stigmatized disabilities. As a highly stigmatized condition with similar social cognitive features to autism, schizophrenia may offer a useful comparison for stigma. Previous studies have found that autistic people may be perceived more favorably than those with schizophrenia, but little is known about the underlying volitional thoughts that contribute to differences in how these conditions are perceived.

**Methods:**

The present study utilizes a mixed-methods approach, allowing for a detailed understanding of how young adults perceive different diagnostic labels. 533 college undergraduates completed questionnaires reflecting their perceptions of one of eight diagnostic labels: four related to autism (autism, autistic, autism spectrum disorder, or Asperger’s), two related to schizophrenia (schizophrenia or schizophrenic), and two related to an unspecified clinical condition (clinical diagnosis or clinical disorder). Participants also completed an open-ended question regarding their thoughts about, and exposure to, these labels. Responses were compared across broader diagnostic categories (autism, schizophrenia, general clinical condition), with thematic analysis used to assess the broader themes occurring within the open-ended text.

**Results:**

While perceptions did not differ significantly for person-first and identity-first language within labels, several differences were apparent across labels. Specifically, quantitative results indicated greater prejudice towards autism and schizophrenia than the generic clinical condition, with schizophrenia associated with more perceived fear and danger, as well as an increased preference for social distance, compared to autism. Patterns in initial codes differed across diagnostic labels, with greater variation in responses about autism than responses about schizophrenia or the general clinical condition. While participants described a range of attitudes toward autism (patronizing, exclusionary, and accepting) and schizophrenia (fear, prejudice, and empathy), they refrained from describing their attitudes toward the general clinical label, highlighting the centrality of a cohesive group identity for the development of stigma. Finally, participants reported a number of misconceptions about autism and schizophrenia, with many believing features such as savant syndrome to be core characteristics of the conditions.

**Conclusion:**

These findings offer a more detailed account of how non-autistic individuals view autism and may therefore aid in the development of targeted programs to improve attitudes toward autism.

## Introduction

Stigma has traditionally been defined as the social discrediting and marginalization that occurs in response to negatively perceived attributes within a prevailing society (1), with more modern conceptualizations emphasizing the lower status and power afforded to stigmatized groups (2, 3). One marginalized group that continues to be stigmatized across many cultures (4–6), despite recent increases in acceptance and awareness (7), is autistic people. Autistic children and adults often behave and communicate in non-normative ways, and these differences are reliably rated by non-autistic observers as less socially appealing (8, 9). Attitudes about autism do vary among non-autistic people (10), with greater autism acceptance occurring among those with more autism knowledge and experience [for a review, see Kim et al. (11)], but non-autistic observers as a whole express a general reluctance to interact with autistic people (9). This process is mitigated somewhat but still persists when raters are informed that the person they are observing is autistic [(12); for a review see, Thompson-Hodgetts et al. (13)], or when they are first educated about autistic differences, neurodiversity, and inclusion (14–17).

Stigma toward autistic differences contributes to the social exclusion of autistic people (18), increases experiences of minority stress (19), affects mental health (20), and impedes personal and professional achievement (13). This stigma can also turn inward among autistic people (19) and contribute to conscious and unconscious concealment strategies to avoid victimization (21) that are mentally and emotionally taxing and associated with poor mental health outcomes (22, 23).

Although the nature, experiences, and consequences of stigma toward autism has received considerable attention (18), less is known about how autism stigma compares to the stigma associated with other specific clinical conditions, or perceptions of clinical conditions more generally. Charting responses to a generic, unspecified clinical condition may provide a baseline to extract aspects of stigma associated with autism, and comparisons to another stigmatized neurodivergent group can specify the aspects of stigma unique to autism that can be used to target prejudice or misconceptions that are specific to autism, inform knowledge-based interventions, and improve education and dissemination of autism-relevant information.

For instance, comparing autism-related stigma to stigma toward another stigmatized condition, schizophrenia, may be instructive. Like autism, schizophrenia is defined in part by social difficulties (24), is misunderstood in the general population (25, 26), and is associated with considerable stigma (27). Unlike autism, however, schizophrenia is often characterized by negatively viewed symptoms such as hallucinations and delusions. Exaggerated and distorted media portrayals (28) have reinforced stereotypes and misperceptions of people with schizophrenia as unpredictable and dangerous (29), which may contribute to fear-based stigma that is less present with autism (30). In truth, research indicates that non-clinical factors such as age and gender are stronger predictors of violence than schizophrenia (31), and those with severe mental illness are more often the victims of violence than the perpetrators of it (32).

Previous research comparing attitudes toward autism and schizophrenia suggests that stigma toward the two conditions often differs, and may relate to knowledge and stereotypes about the conditions (30). While the majority of adults can recognize the terms autism and schizophrenia, far fewer can accurately describe their characteristics (33). In particular, non-autistic people show large variability in their understanding of the causes, age of onset, and need for lifelong treatment associated with an autism or schizophrenia diagnosis (33), but tend to believe that autistic people are more capable of living a “normal” life than those with schizophrenia. In line with greater functional assessments, non-autistic people also report less stigma toward autism (34), perceiving them as intelligent and creative, while people with schizophrenia were more likely to be perceived as dangerous (30). The increased severity of stigma toward people with schizophrenia extends to social attitudes; while non-autistic people report a reluctance to interact with both autistic people and people with schizophrenia (30), this stigma is more severe and wider ranging for hypothetical interactions with a person with schizophrenia, extending to familial, workplace, and educational settings (33).

Most studies of stigma have used quantitative measures and scales to assess attitudes about autism and schizophrenia. Such approaches are useful for comparing individuals and groups of people on a uniform set of items, but they restrict responses to pre-determined questions and therefore may fail to fully capture conscious feelings. Complementing quantitative assessments with qualitative analysis in which participants describe clinical conditions in their own words allows for a richer view of how conditions are perceived. For instance, qualitative data can be used to assess specific patterns in how people stigmatize a condition instead of just quantifying degrees of stigma, help inform the underlying volitional thoughts that drive quantitative results, and determine whether emergent themes are consistent with survey data.

The present study utilizes a mixed-method approach that incorporates both self-report questionnaires and open-ended responses to understand how adults perceive autism-related diagnostic labels compared to schizophrenia-related labels, as well as general clinical labels. We hypothesized that participants would endorse more negative attributes and attitudes toward autism-related labels and a lower willingness to interact with individuals with these labels, compared to labels associated with a general diagnostic condition. However, based on previous data showing high levels of stigma toward individuals with schizophrenia, particularly around misperceptions (35, 36), as well as findings that autistic people are perceived more positively when they are labeled as autistic compared to when they are labeled as having schizophrenia (12), we hypothesized that autism-associated labels would be perceived more favorably than schizophrenia-associated labels.

It is also possible that differences in how conditions are described can affect stigma, with certain labels increasing the salience of difference, disability, or severity than others. Therefore, a secondary aim was to determine whether perceptions of clinical conditions vary between person-first (e.g., person with autism) and identity-first labels (e.g., autistic person) within diagnostic categories, as well as between different labels within the autism spectrum (e.g., Asperger’s, on the spectrum). In general, autistic adults tend to prefer identify-first language, while professionals and practitioners continue to favor person-first language (37, 38). Within the diagnostic conditions, we expected more positive perceptions for the “person with Asperger’s” label relative to other autism-related labels, as some findings have suggested that the Asperger’s label is associated with less stigma and considered less severe compared to the autism label (39). We also explored whether person-first labels (“person with schizophrenia”) would be perceived more positively than identity-first labels (“schizophrenic person”) within the schizophrenia condition, as would be predicted by proponents of person-first language who seek to separate the person from a highly stigmatized condition (40). There is a lack of consensus on preferred technology among people with severe mental illness, with individual preferences often varying across situations and contexts (41), while clinicians often encourage person-first language in an effort to reduce stigma (42). However, because person-first language in practice is typically restricted to conditions that are stigmatized, such efforts may backfire and unintentionally accentuate stigma rather than reduce it (43). While previous literature has suggested an association between identity-first language and greater stigma (44), more recent findings suggest that it is the lack of an explicit diagnostic label, rather than the phrasing of the label, that elicits the greatest impact on stigma (45, 46). In particular, vignettes of people with schizophrenia were associated with greater fear, anger, blame, and perceived danger when a diagnostic label was not provided, while these responses did not differ significantly when person-first and identity-first labels were used (46). Likewise, videos of autistic adults are typically rated more favorably when the person’s diagnosis is disclosed (12), regardless of whether person-first or identity-first language is used (10).

In order to emphasize a data-driven approach, no specific *a priori* hypotheses were generated for the qualitative portion of this study, though we expected differences in the themes used to describe diagnostic labels for autism, schizophrenia, and a general diagnosis.

## Materials and methods

### Participants

A total of 533 college undergraduates aged 18–63 (*M* = 21.22, SD = 5.67) were recruited from the University of Texas at Dallas. Four participants with an IQ below 80, as estimated by the Reading subtest of the Wide Range Achievement Test 3 [WRAT3; (47)], were excluded from analysis, resulting in a final sample of 529 participants (*M*_*IQ*_ = 109.26). Participants were predominantly female (78%) and a plurality were White (43%), with the remaining participants identifying as Asian (40%), Black (7%), American Indian/Alaska Native (1%), or other races (9%). To better approximate a general population sample, participants were not screened for psychological or psychiatric conditions. Participants received course credit for their participation. All aspects of this protocol were approved by the UT Dallas Institutional Review Board.

### Procedure

After providing informed consent, participants completed the WRAT3 and several computerized questionnaires assessing their perceptions of different diagnostic labels, along with an open-ended question regarding their thoughts about, and exposure to, each label. Participants were randomly assigned to a survey condition in which the wording of each measure was modified to feature one of eight labels: four related to autism (AUT; person with autism, person with autism spectrum disorder, person with Asperger’s, or autistic person), two related to schizophrenia (SCZ; person with schizophrenia or schizophrenic person), and two related to a general clinical condition (CDX; person with a clinical diagnosis or person with a clinical disorder). These labels enabled the comparison of perceptions of: (1) person-first relative to identity-first language; (2) autism-related labels to a distinct clinical condition also characterized by social difficulties, schizophrenia; and (3) autism and schizophrenia labels to a more general clinical condition. The inclusion of the general clinical label allowed us to determine whether autism and schizophrenia are perceived more positively or negatively relative to the invocation of clinical diagnoses more generally. Due to the larger number of autism-related labels relative to schizophrenia and general labels, the sample size of participants assigned to autism-related labels was larger than that of the other two categories (*N*_*AUT*_ = 269, *N*_*SCZ*_ = 129, and *N*_*CDX*_ = 130).

### Measures

#### Stigma questionnaires

##### The attributes and reactions scale (AAR)

The AAR (35) is a five-point Likert scale originally designed to measure attitudes toward schizophrenia and depression. In the first part of this measure, participants were given eight stigma-related behavioral attributes (unpredictable, lacking self-control, aggressive, frightening, dangerous, needy, dependent on others, and helpless) and asked to rate the extent to which each attribute applied to a person with the assigned diagnostic label, with higher scores indicating greater agreement. Due to a technical error, ratings for “aggressive” were not collected. Responses were averaged across two subscales (35): perceived dangerousness and perceived dependency.

For the second portion of this measure, participants were given nine emotional labels (fear, uneasiness, feelings of insecurity, pity, empathy, desire to help, anger, ridicule, and irritation) and asked to indicate the degree to which they would respond in such a way toward a person with the given label, with higher scores indicating a more likely emotional reaction. Responses were averaged to form three subscales (35): fear, pity, and anger. The behavioral attributes portion of this scale showed strong internal consistency across all diagnostic categories (α_*AUT*_ = 0.819, α_*SCZ*_ = 0.841, and α_*CDX*_ = 0.811), while the emotional reactions portion showed lower, but acceptable internal consistency (α_*AUT*_ = 0.701, α_*SCZ*_ = 0.635, and α_*CDX*_ = 0.648).

##### The prejudice scale

The Prejudice Scale (36) is an 18-item scale developed to measure negative attitudes toward schizophrenia. The scale was adapted in this study by replacing “schizophrenia” with each of the eight tested labels. Participants were shown items reflecting prejudiced attitudes toward the assigned label [e.g., “(Label) patients/patients with (label) should be kept in hospitals”] and answered either “I agree” or “I disagree” for each statement. Scores of either 1 or 2 were assigned to each response based on agreement or disagreement, with higher scores indicating more negative attitudes toward the label. A total score was generated by summing individual item scores (36), with possible scores ranging from 18 to 36. Internal consistency for the overall measure was low to modest across groups (α_*AUT*_ = 0.433, α_*SCZ*_ = 0.610, and α_*CDX*_ = 0.426).

##### The social distance scale (SDS)

The SDS (48) is a six item Likert scale developed to assess stigma toward autistic individuals and adapted here to include each of the eight labels. Participants were shown 6 items assessing their willingness to form hypothetical social relationships with a person with each label [e.g., “How willing would you be to make friends with a person with (label)?”]. Responses were scored on a four-point Likert scale, with higher scores indicating a lower willingness to interact. Scores on each item were summed to form a composite score, with possible scores ranging from 6 to 24. This measure showed strong internal consistency within each diagnostic category (α_*AUT*_ = 0.824, α_*SCZ*_ = 0.803, and α_*CDX*_ = 0.811).

#### Open ended question

Participants were also given an open-ended question about their assigned label, which formed the basis for a qualitative analysis. Participants were asked “what do you think of when you hear the word(s) (label)?” Open-ended questions were administered privately via computer, and participants were given as much time to respond as needed. Responses ranged from 0 to 788 characters in length, with a mean length of 136.34 characters (SD: 111.03), and eight participants providing no response.

### Analysis plan

#### Quantitative analysis

Preliminary one-way ANOVAs were conducted to determine where person-first and identity-first labels were perceived differently within each diagnostic condition. Summary scores on each of the three stigma questionnaires did not differ significantly between any of the 4 labels within the autism-associated labels, nor did they differ between the 2 schizophrenia-associated labels or between the 2 general clinical diagnosis labels (Supplementary Figures 1–3). Subsequent analyses therefore were conducted at the level of diagnosis (i.e., labels within each of the three diagnostic conditions were combined). A one-way ANOVA was then used to assess whether summary scores on the seven stigma measures differed for autism, schizophrenia, and a general clinical condition. Significant findings were followed up with *post hoc* Tukey tests for multiple comparisons. Due to the large number of analyses performed, the significance cutoff was adjusted to 0.01.

#### Qualitative analysis

Because quantitative results did not differ between the different labels for each condition, qualitative analyses focused on each condition collapsed across labels (for an overview of the most common codes for all 8 labels, see Supplementary Table 1). Specifically, a thematic analysis was conducted to gain further insight concerning perceptions of autism, schizophrenia, and a general clinical condition, and to highlight differences in how participants describe these conditions. This method was chosen due to its ability to provide a rich analysis of large qualitative datasets, as well as its recent use within autism research (49, 50). Responses to open-ended questions were aggregated and themes were identified and coded by the first author based on the six-step approach to thematic analysis outlined by Braun and Clarke (51, 52). First, all responses were given an initial read-through by the first author to gain familiarity with the data prior to coding. Next, initial codes for the data were generated in a data-driven fashion using QDA Miner (53). Initial codes were created by identifying frequently occurring patterns of responses across the dataset. Similar codes were then clustered and organized into themes and sub-themes, which were further refined and simplified to eliminate any redundancies and minimize overlap. Once finalized, themes were named based on the shared narrative they conveyed. Codes, themes, and sub-themes were reviewed by both authors, and consensus was reached for any areas of disagreement.

## Results

### Quantitative analysis

Means and Standard deviations (SDs) for the three label conditions are presented in Table 1. On the AAR, perceptions of danger differed significantly between diagnostic labels [*F*_(2,526)_ = 19.77, *p* < 0.001], with greater danger attributed to schizophrenia compared to autism (*p* < 0.001) and a general clinical condition (*p* = 0.002). A similar pattern was found for fear [*F*_(2,526)_ = 22.56, *p* < 0.001], with participants reporting greater feelings of fear in response to schizophrenia compared to autism and a general clinical condition (*ps* < 0.001). Perceived dependency did not differ significantly across diagnostic conditions [*F*_(2,526)_ = 1.63, *p* = 0.197], nor did feelings of pity or anger (*ps* > 0.417).

**TABLE 1:** Means and standard deviations (SD) for measures of stigma.

	Autism labels	Schizophrenia labels	General clinical labels
Perceived danger	2.42 [0.71]	2.93 [0.88]	2.61 [0.73]
Perceived dependency	2.77 [0.84]	2.75 [0.79]	2.91 [0.81]
Fear	2.05 [0.88]	2.68 [0.95]	2.11 [0.85]
Pity	3.58 [0.69]	3.56 [0.78]	3.66 [0.71]
Anger	1.34 [0.58]	1.35 [0.67]	1.37 [0.56]
Prejudice	22.10 [2.04]	22.07 [2.48]	21.27 [1.92]
Social distance	11.50 [3.33]	12.97 [3.37]	11.66 [3.28]

Groups differed significantly for scores on the Prejudice Scale, [*F*_(2,526)_ = 7.35, *p* = 0.001]. Both schizophrenia (*p* = 0.007) and autism (*p* = 0.001) were associated with more prejudice than the general clinical condition, but prejudice did not differ significantly between schizophrenia and autism (*p* = 0.988). However, many group differences were present at the individual item level for this measure, with participants believing that people with schizophrenia are more dangerous, untrustworthy, and pose greater harm to children, and reporting greater opposition to having a relative marry a person with schizophrenia, compared to an autistic person (*p*s < 0.001) or a person with a general clinical condition (*p*s < 0.01). Compared to schizophrenia, participants also believed that autism was more visibly detectable, less likely to require hospitalization, had greater potential for treatment and recovery, and benefited more from psychotherapy and pharmacological interventions, and showed more favorable attitudes toward having an autistic neighbor (*p*s < 0.01). On the SDS, schizophrenia was associated with greater stigma [*F*_(2,526)_ = 8.97, *p* < 0.001], with participants endorsing a significantly larger social distance to schizophrenia relative to both autism (*p* < 0.001) and a general clinical condition (*p* = 0.005).

### Qualitative analysis

#### Autism

A total of 88 initial codes were generated for autism, with each code occurring in <1–15% of cases. These codes reflected a wide range of perceptions, with the most frequently occurring codes (Table 2) focusing on poor social abilities (15%), a range of severities (10%), and dependence on others (10%). Initial codes and their context within the data were used to generate four themes: (1) perceived severity, (2) symptoms or features, (3) attitudes toward autism, and (4) representations of autism. Each theme was further divided into subthemes. Full themes and subthemes, along with representative responses, are reported in Table 3.

**TABLE 2:** Frequently occurring initial codes.

Code	% cases
**Autism labels (*N* = 269 cases)**	
Poor social abilities	15.20%
Range of severities	10.40%
Dependent on others	10.00%
Intelligent	8.10%
Intellectual disability	7.80%
Misunderstand social cues	7.00%
Awkward	6.70%
General deficits	5.90%
General mental illness	5.60%
Difficulties understanding emotions of others	5.60%
**Schizophrenia labels (*N* = 129 cases)**	
Psychotic symptoms	38.00%
General mental illness	19.40%
Needs treatment	13.20%
Multiple personalities/dissociative	8.50%
Emotional instability	7.80%
Can be managed	7.80%
Unfairly stigmatized	6.20%
Crazy	5.40%
Brain disorder	5.40%
Can live a “normal life”	5.40%
**General condition labels (*N* = 130 cases)**	
Brain or behavioral conditions	63.10%
Autism	17.70%
Impaired functional abilities	15.40%
Depression	13.80%
Broad range/spectrum	13.80%
Diagnosed by a doctor	13.10%
Schizophrenia	12.30%
Needs treatment	8.50%
Anxiety	6.90%
Mania/bipolar disorder	6.20%
Dependent on others	6.20%
Personality disorder	5.40%
Developmental disability	5.40%
Physical impairment	5.40%
Not normal	5.40%

Text-based responses to an open-ended question about perceptions toward labels related to autism (ASD), schizophrenia (SCZ) or a general clinical condition (CDX) were analyzed using thematic analysis. Initial codes reported in greater than five percent of cases (*N_AUT_* = 15 cases, *N_SCZ_* = 7 cases, and *N_CDX_* = 7 cases) are reported for each category. Initial codes were used to help generate themes and subthemes within the data.

**TABLE 3:** Themes and subthemes within open-ended responses for autism labels, with text examples.

Theme	Subtheme	Quoted example from responses
Perceived severity	Severe disability	“A person whose mental ability is limited and they rather depend on their parents.”
	Minimal disability	“I think of a disorder that a person is born with. It doesn’t mean that the person is less intelligent or strange. It just means that their brains make connections differently than most people’s do.”
	Range of presentations	“A spectrum of disability that mostly deals with communication skills, but also impacts other aspects of life. Can be incredibly mild, or require life-long care.”
Symptoms or features	Social difficulties	“When I hear the word “autistic,” I generally tend to think of people who have a hard time connecting to other people in a permanent fashion. They want to create bonds with other people but just don’t know how to create and maintain these relationships”
	RRBIs	“I believe each person with autism has unique characteristics where they can fixate on one thing or have a specified routine everyday. it does not seem that they like change”
	Emotion and behavior dysregulation	“Cannot understand their own inward emotions from time-to-time and act on them appropriately. This may make the autistic person seem a bit impulsive or compulsive”
	Speech and communication difficulties	“Disorder that doesn’t allow you to process or communicate the same way others do.”
	Savant syndrome	“Have extraordinary memory about what they did or saw (such as immediately memorizing road signs or car types or people’s names with faces).”
Attitudes toward autism	Exclusionary attitudes	“They seem to be more social but tend to not be always be wanted in social setting. They make people feel uneasy and not comfortable to be around”
	Patronizing attitudes	“Wants to be accepted and loved. But doesn’t understand how to be accepted. Someone who wants to be just like everyone else.”
	Autism acceptance	“I don’t subscribe to the harmful stigmas associated with autism nor am I very fond of them, mostly because they dehumanize individuals who, at the end of the day, are human. Yes, their cognitive processes may be different, but that doesn’t necessarily make them any less intelligent or any more piteous than people without autism.”
Representations of autism	Personal contact	“I think of two people from high school, one whom I was great friends with, and one that was seen as an outcast of sorts.”
	Media	“The famous woman who is autistic who was known for building the cattle processing machine that became something used nationwide in many farms.”

#### Schizophrenia

Open-ended responses for schizophrenia labels were used to generate 86 initial codes, which appeared within <1–38% of cases. Psychotic symptoms (38%), general mental illness (19%), and the need for treatment (13%) were among the most frequently occurring codes (Table 2). These codes and their associated responses centered around three general themes: (1) schizophrenia knowledge, (2) attitudes toward schizophrenia, and (3) representations of schizophrenia. These themes and their respective subthemes, along with representative responses, are reported in Table 4.

**TABLE 4:** Themes and subthemes within open-ended responses for schizophrenia labels, with text examples.

Theme	Subtheme	Quoted example from responses
Schizophrenia knowledge	Accurate knowledge of symptoms, cause, and prognosis	“Treatable psychological disorder with potential delusions and/or hallucinations.”
	Misperceptions of symptoms and cause	“When I hear the word schizophrenic I think of someone who has multiple personalities but is highly intelligent at one specific topic/interest.”
Attitudes toward SCZ	Understanding and empathy	“They are not mean, dangerous people, but people who need to be helped and treated the way everyone should be treated.”
	Fear or perceived danger	“I think violent, unpredictable, and intelligent mainly.”
	Prejudice toward mental illness	“My first thought is ‘crazy’.”
Representations of SCZ	Personal contact	“My uncle. He was the only person who I had regular interactions with and he was diagnosed with schizophrenia during adolescence.”
	Media	“I think of the TV show Criminal Minds because one of the characters mothers has schizophrenia.”
	Classroom exposure	“When I hear schizophrenic, I think of a mental disorder that I learned about in psychology.”

#### General clinical condition

Participant responses for general clinical labels produced 62 initial codes, which appeared within 1 to 63% of cases. References to brain or behavioral conditions (63%), autism (18%), and impaired functional abilities (15%) were among the most frequent codes for these labels. These codes and the responses in which they occurred were used to generate three broader themes: (1) clinical conditions, (2) symptoms or features, and (3) clinical pathology. These themes were divided into subthemes, which are reported in Table 5, along with representative responses.

**TABLE 5:** Themes and subthemes within open-ended responses for general clinical labels, with text examples.

Theme	Subtheme	Quoted example from responses
Clinical conditions	Psychological conditions	“Any diagnosis from a psychiatrist in regards to normal brain health, e.g., autism, schizophrenia, mania, manic depression, MDD, etc.”
	Physical disabilities	“Possesses lacking physical capabilities that undermine normal bodily expression.”
Symptoms or features	Impaired adaptive skills	“I think of a condition upon an individual that presents a disadvantage to them in social, academic, and work related contexts in comparison to other individuals.”
	Cognitive difficulties	“Somebody who has an impairment that limits their ability to “normally” respond to stimulus around the world. This can mean somebody who doesn’t understand others, or somebody who doesn’t respond to situations “normally,” whatever that could mean in some situation.”
Clinical pathology	Causes	“Clinical diagnosis’ in my opinion are the result of stressful events in collaboration with genes, thus both have a hand to play in the onset of many disorders, but not all.”
	Treatment	“I think of a disorder that requires treatment of some sort in order for the individual to maintain homeostasis within the realms in their lives.”
	Prognosis	“Is able to live a normal life depending on how they learn how to adapt.”

## Discussion

The current study used a mixed-methods approach to compare stigma between autism, schizophrenia, and a generic clinical condition. Quantitative results indicated greater prejudice toward autism and schizophrenia than the generic clinical condition, with schizophrenia differentiated from autism by being associated with perceptions of danger and fear, and a greater preference for social distance than from autism. Qualitative results supported and expanded these findings, with autism described in greater depth but with less cohesion than the other conditions, and schizophrenia more commonly described with references to danger and more frequent uses of derogatory terms for mental illness.

A secondary aim of the study was to compare stigma toward clinical conditions labeled with person-first language (e.g., “person with autism”) relative to identity-first language (e.g., “autistic person”). Language choices for autism and other clinical conditions are often intensely debated and discussed, as use of person-first versus identity-first language can reflect ideological differences that may affect conceptualizations and biases (54). However, preliminary examination indicated that stigma did not differ between person-first and identity-first language, either for autism or schizophrenia, nor did it differ between different labels of the autism spectrum (“autism,” “on the spectrum,” “Asperger’s”), and thus subsequent quantitative analyses were pursued after collapsing across person-first and identity first-labels. The lack of a language effect here aligns with a previous study that found that referring to autistic people with person-first or identify-first language does not affect the first impressions they receive from non-autistic observers (12), as well as research suggesting that it is the lack of a label, rather than person-first or identity-first language, that most contributes to stigma toward schizophrenia (46). Broader language use surrounding disability, however, still influences perpetuation of stigma in other ways (54), and it is possible that the use of person-first and identity-first language may have differing effects on those to whom the label is attributed Because preferred terminology has been the subject of recent debate within disability communities, with the preferences of disabled people differing from those of clinicians and stakeholders (55, 56), future research examining the effect of person-first and identity-first language on self-esteem and internalized stigma may offer greater explanation to the potential impact of differing terminologies.

Although stigma did not differ among labels within each clinical condition, it did differ between the conditions themselves in a number of ways. First, autism and schizophrenia were ascribed more stigma than a generically labeled clinical condition. This suggests that autism and schizophrenia are more stigmatized conditions than clinical conditions are generally, with each exceeding the baseline of stigma attributed to a non-specified clinical status. However, stigmatizing attitudes may also be generated to a greater degree when a specific diagnostic label is provided compared to a generic one. Encountering the terms “autism” or “schizophrenia” may trigger certain stereotypes, encourage consideration of specific knowledge and (mis)information, and inspire personal reflection on experiences with each condition. In contrast, a generic clinical label may not be associated with specific enough experience or information to generate high levels of stigma. Supporting this interpretation, the thematic analysis of the qualitative data indicated that the general clinical condition did not elicit as many discernable attitudes or references to first-hand exposure as did participant descriptions of autism and schizophrenia. This suggests that discrete clinical labels may play a central role in the development of stigma by providing categories that can be linked to specific attitudes, information, and biases.

Second, both quantitative and qualitative analyses suggested important differences in the stigma ascribed to autism and schizophrenia. Descriptions of autism were more detailed and wide-ranging than those of schizophrenia, covering more concepts, characteristics, and attitudes. This may reflect greater familiarity with autism than schizophrenia, both interpersonally and from broader cultural messaging, awareness campaigns, and media portrayals. Given comparable prevalence estimates for autism and schizophrenia of about 1% (57, 58)−at least until recent reported increases for autism (59)−greater familiarity with autism is likely not driven by substantially higher cases of autism within communities than schizophrenia. Rather, it may reflect increasing awareness, acceptance, and inclusion of autism that has not occurred to the same degree for schizophrenia (60). This disparity in stigma may also be echoed in diagnostic disclosure decisions. Although disclosing one’s autism can be a fraught decision for many autistic people that often depends on many internal and external factors (61), disclosure of schizophrenia may be especially perilous given rampant biases, beliefs, and misinformation about the condition (62). Disparities in disclosure between the two conditions may in some respects be a reflection of disparities in the stigma attached to them.

Over time, increases in disclosure can demystify clinical conditions, facilitate personal exposure, and help reduce stigma (63), yet findings from this study underscore reasons people may be hesitant to disclose a schizophrenia diagnosis. Although misconceptions were common for both autism and schizophrenia, with wide variability in participant understanding and acceptance of autism, schizophrenia was uniquely characterized by misinformation concerning propensity for violence and disparaging comments about mental illness. This pattern was especially prevalent within the quantitative data, with participants reporting similar levels of pity, anger, and perceived dependency toward both autism and schizophrenia, but associating schizophrenia with significantly greater fear, perceived danger, untrustworthiness, and potential to harm children. Perceived danger, which is more frequently attributed to schizophrenia than other conditions (33), is a strong predictor of stigma (64) and may underlie the persistent stigma attached to schizophrenia (65). Indeed, while overall prejudice did not differ significantly between conditions, participants were more hesitant to interact with a hypothetical person with schizophrenia than an autistic person, even for less intimate interactions. This is consistent with previous literature reporting greater social distance for schizophrenia than autism (30, 33), and highlights the need for improved education and targeted initiatives to counter stigma about schizophrenia (66).

This study has several limitations that should be considered when interpreting the findings reported here. Most notably, the sample−while large−consisted exclusively of university students, who generally are more inclusive and progressive about disability and mental health differences than the general population (67, 68). Participants were also racially diverse, and younger and more educated than the general population. Because schizophrenia is often diagnosed in early to mid-adulthood (57), while autism is typically diagnosed in childhood (69), it is possible that participants may have had less exposure to schizophrenia than autism. However, many participants in both the autism and schizophrenia conditions described personal contacts with both autistic people and people with schizophrenia, and literature suggests that reported contact with autistic people and people with schizophrenia may occur at similar rates in adults (33). Therefore, it is uncertain whether exposure to the two conditions may have impacted the reported findings. Participants were recruited through the university’s psychology research pool, meaning that the majority of these individuals were actively enrolled in a psychology course and may have been exposed to ideas about autism and schizophrenia through their coursework. This may explain the unexpected, but relatively frequent references to autism and schizophrenia in responses to the control labels. Thus, the stigma reported here for autism and schizophrenia may not reflect stigma in the population more broadly, and may be a conservative estimate of stigma that would be found in a more representative sample.

While the current study was underpowered to test for differences based on participant age, it is possible that these results may not generalize to an older sample of raters, and future research should aim to characterize the effects of age on perceptions of autism and schizophrenia. Further, this study should not be taken as a comprehensive examination of attitudes and beliefs about autism and schizophrenia. Although the mixed method approach used here provided deeper and more nuanced information concerning the nature of stigma attributed to the two conditions, the measures used were limited, and the lack of inclusion of other clinical conditions precluded the opportunity to examine how autism and schizophrenia are perceived relative to other types of disability and conditions. A within-subjects comparison of quantitative and qualitative accounts of stigma toward these labels may offer greater clarity regarding the relationship between one’s perceptions of a label and their associated social attitudes.

These limitations notwithstanding, the current study provides quantitative and qualitative evidence that attitudes about autism and schizophrenia vary widely among participants. Although many emphasized the importance of understanding, acceptance, and inclusion, negative attitudes and misinformation were also common. In particular, results indicated that schizophrenia remains a highly stigmatized condition, driven primarily by participants’ beliefs concerning danger and fear that was not present in attitudes about autism. Countering these misconceptions, reducing stigma, and promoting inclusion should be important priorities for educators and clinical practitioners.

## Data availability statement

The datasets presented in this study can be found in online repositories. The names of the repository/repositories and accession number(s) can be found below: https://osf.io/3k6zj/?view_only=681901d6f3f44de1a5da2f021ce4f41b.

## Ethics statement

The studies involving humans were approved by the University of Texas at Dallas Institutional Review Board. The studies were conducted in accordance with the local legislation and institutional requirements. The participants provided their written informed consent to participate in this study.

## Author contributions

DJ: Conceptualization, Formal analysis, Funding acquisition, Methodology, Visualization, Writing−original draft, Writing−review and editing. NS: Funding acquisition, Project administration, Resources, Supervision, Writing−review and editing, Writing−original draft.
