# Supplementary figures and images for: A mixed method comparison of stigma toward autism and schizophrenia and effects of person-first versus identity-first language
Source: Front Psychiatry. 2023 Oct 27;14:1263525. doi: 10.3389/fpsyt.2023.1263525 (PMC10641499; doi:10.3389/fpsyt.2023.1263525)

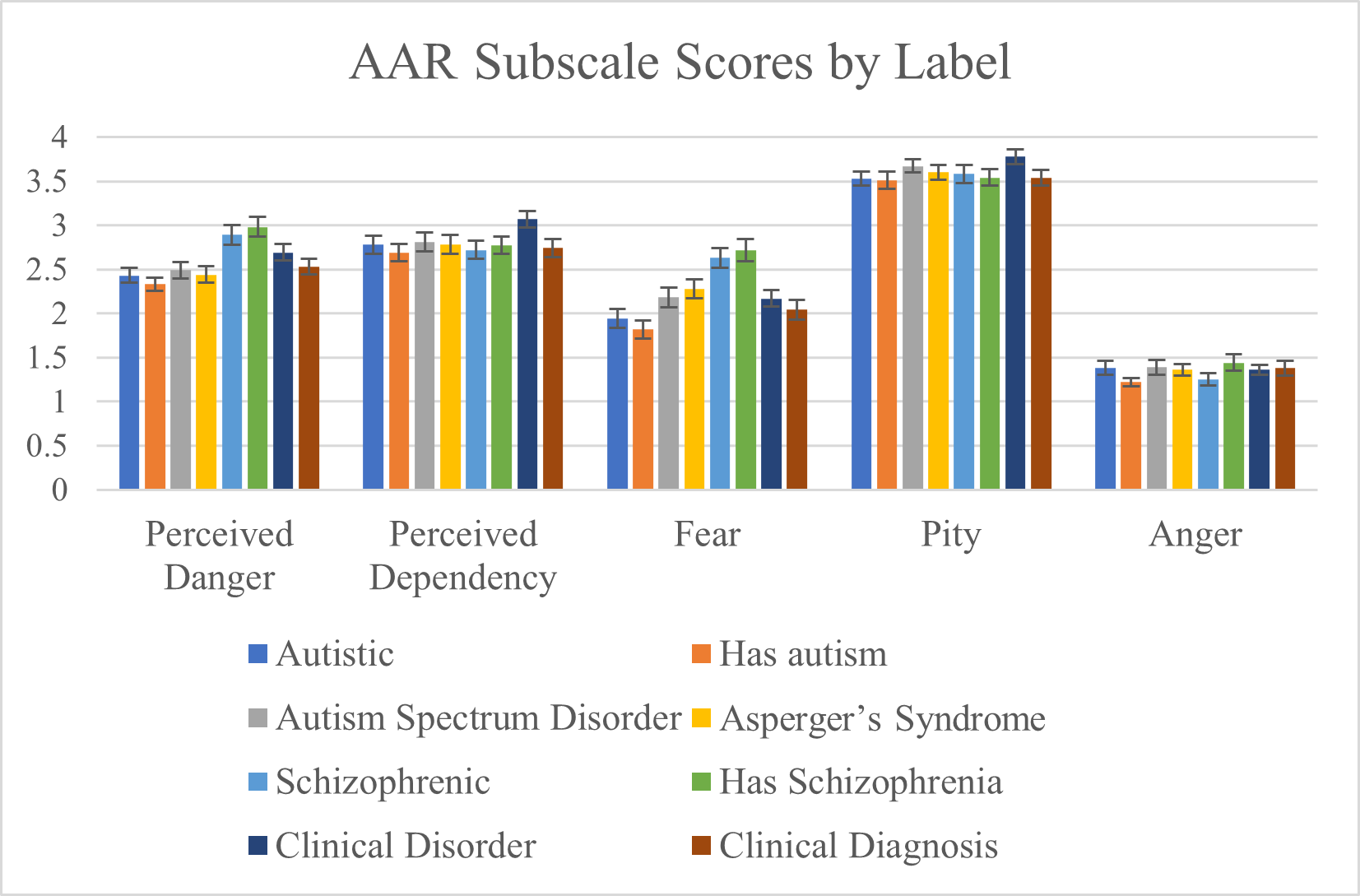

Supplement: Supplementary file 2 [file Image_1.PNG]

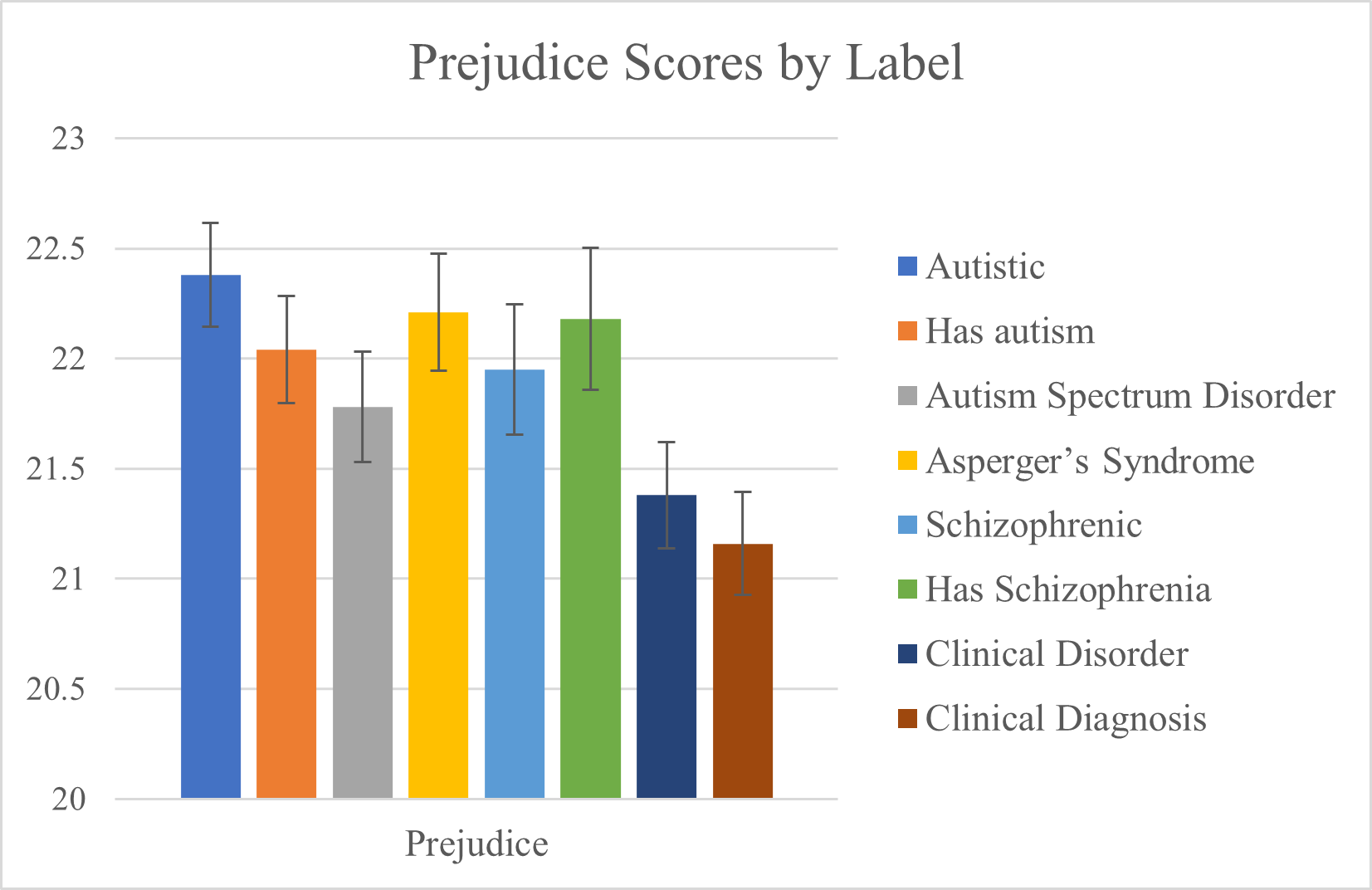

Supplement: Supplementary file 3 [file Image_2.PNG]

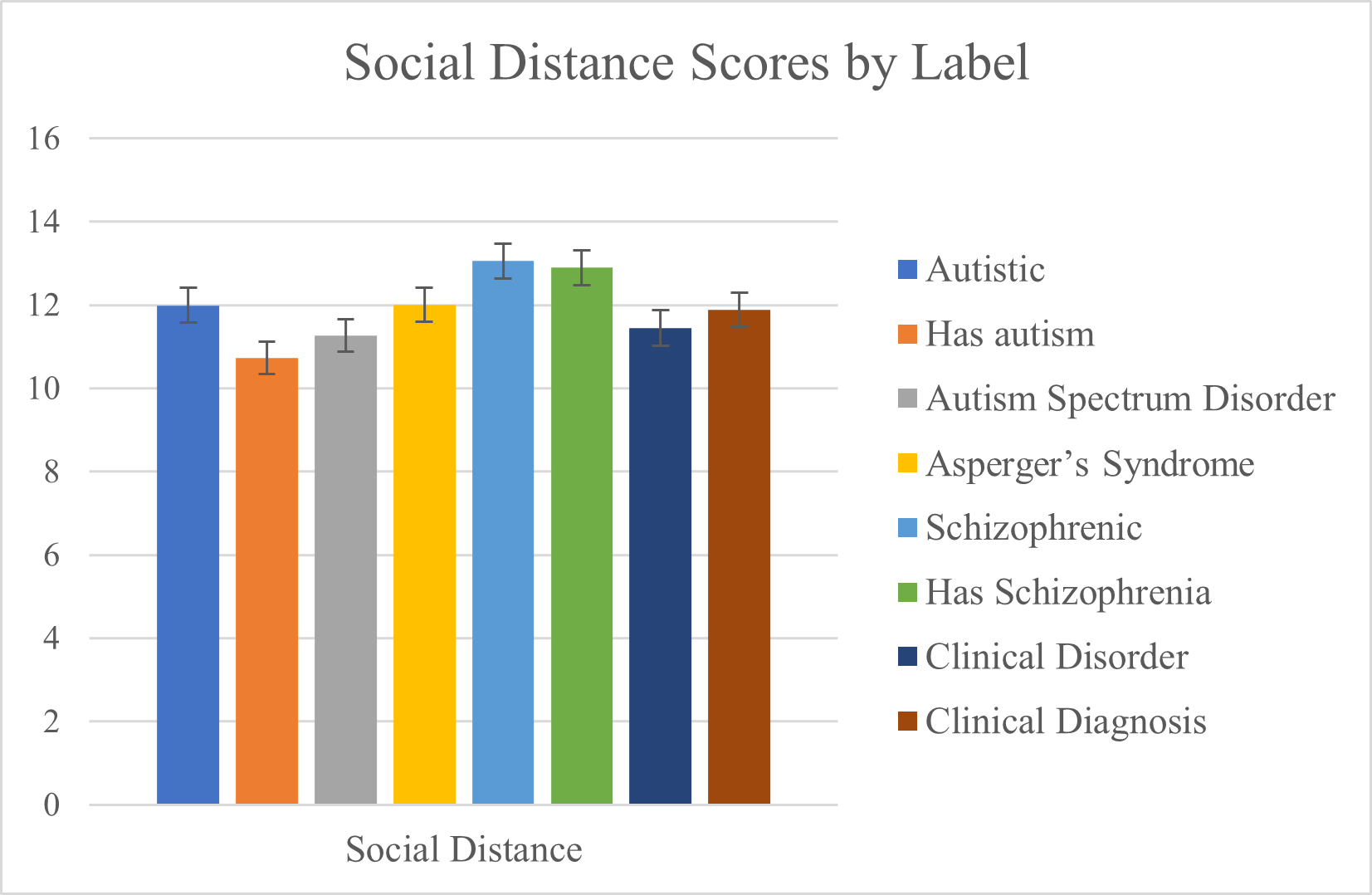

Supplement: Supplementary file 4 [file Image_3.PNG]
